# Size matters: Aging in a Retirement Metropolis

**DOI:** 10.1093/geroni/igaf122.395

**Published:** 2025-12-31

**Authors:** Galit Nimrod

**Affiliations:** Ben Gurion University of the Negev, Be’er Sheva, HaDarom, Israel

## Abstract

Despite the abundance of retirement communities in the U.S., none compares to The Villages in Central Florida. Home to nearly 150,000 residents, it is the world’s largest retirement community and the only one that can be considered a city for older adults. Based on in-depth interviews with 40 residents and two months of participant observations, this study explored (among other things) why people move there and their perceptions of its unprecedented size. Results indicated that while some residents resent the place’s growth and the problems that it causes (e.g., traffic, crowdedness), The Villages’ size yields its most attractive qualities: the number and variety of leisure activities, the vast population of like-minded people, and the value for money. Residents perceive a “typical villager” as active, fun-oriented, friendly, down-to-earth, and “young.” These characteristics and study participants’ identification and appreciation thereof point to a strong “place identity” among the villagers. To some extent, these characteristics are associated with the primary pull factors: The active and fun-oriented attributes are related to the variety of activities, and the down-to-earth casual trait is connected to the value for money. In addition, all qualities, especially the friendliness and the “young” spirit, describe the like-mindedness of the people. As the pull factors are associated with The Villages’ size, it may be argued that the villagers’ collective place identity indirectly results from the city’s size. It is thus suggested that retirement cities may cultivate individual and collective place identity, contributing to residents’ sense of belonging and pride.

